# Breaking the clip for cargo unloading from motor proteins: mechanism and significance

**DOI:** 10.15698/mic2022.06.779

**Published:** 2022-05-19

**Authors:** Keisuke Obara, Takumi Kamura

**Affiliations:** 1 Graduate School of Science, Nagoya University, Furo-cho, Chikusa-ku, Nagoya 464-8602, Japan.

**Keywords:** Mitochondria, myosin, Mmr1, ubiquitin, proteasome, proteolysis, homeostasis, Saccharomyces cerevisiae

## Abstract

The mitochondrion is an essential organelle involved in ATP generation, lipid metabolism, regulation of calcium ions, etc. Therefore, it should be inherited properly by newly generated cells. In the budding yeast *Saccharomyces cerevisiae*, mitochondria are passed on to daughter cells by the motor protein, Myo2, on the actin cable. The mitochondria and Myo2 are connected via the adaptor protein Mmr1. After reaching daughter cells, mitochondria are released from the actin-myosin machinery and move dynamically. In our recent paper (Obara K *et al.* (2022), Nat Commun, doi:10.1038/s41467-022-29704-8), we demonstrated that the regulated proteolysis of Mmr1 is required for the unloading of mitochondria from Myo2 in daughter cells. Sequential post-translational modifications of Mmr1, *i.e.,* phosphorylation followed by ubiquitination, are essential for Mmr1 degradation and mitochondrial release from Myo2. Defects in Mmr1 degradation cause stacking and deformation of mitochondria at the bud-tip and bud-neck, where Myo2 accumulates. Compared to wild-type cells, mutant cells with defects in Mmr1 degradation possess an elevated mitochondrial membrane potential and produce higher levels of reactive oxygen species (ROS), along with hypersensitivity to oxidative stress.

## MECHANISM OF MITOCHONDRIA UNLOADING FROM MYOSIN DURING BUDDING

**FIGURE 1:**
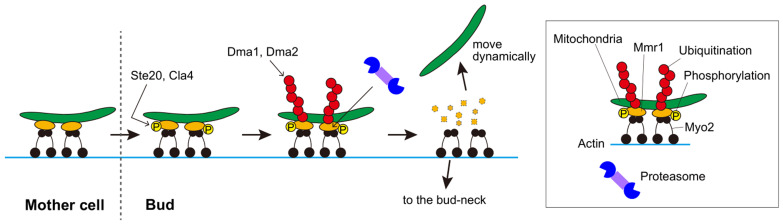
FIGURE 1: Mechanism of mitochondria release from myosin during budding Mitochondria are transported into the growing bud by a type-V myosin Myo2 on the actin cable. Mmr1 bridges mitochondria and Myo2. After mitochondria enter the bud, Mmr1 is phosphorylated, most likely at the S414 residue, by Ste20 or Cla4. Phosphorylated Mmr1 is ubiquitinated by redundant E3 ubiquitin ligases Dma1 and Dma2. Polyubiquitinated Mmr1 is degraded by the proteasome, resulting in dissociation of mitochondria from Myo2. The released mitochondria move dynamically in the bud while Myo2 translocates to the bud-neck to play a role in cytokinesis. As Ste20 and Cla4 are mostly localized to the bud cortex region and nearly absent in the mother cell, phosphorylation of Mmr1 and its turnover take place only after the mitochondria reaches the bud, which may be the mechanism to ensure mitochondrial inheritance to daughter cells.

In *Saccharomyces cerevisiae*, mitochondria are carried to and inherited by daughter cells via type-V myosin Myo2 on the actin cable. Myo2 does not directly bind to mitochondria, but an adaptor protein, Mmr1, bridges mitochondria and Myo2. Upon arrival at daughter cells, mitochondria are released from the actin-myosin machinery and move dynamically in the daughter cell. The mechanism and significance of mitochondrial unloading from myosin remains unknown. Comprehensive analyses have revealed that Mmr1 is a short-lived protein. First, we confirmed that Mmr1 has a short half-life of approximately 20 min. The rapid degradation of Mmr1 by the ubiquitin-proteasome system is responsible for its short lifespan. *In vivo* and *in vitro* experiments demonstrated that the redundant E3 ubiquitin ligases, Dma1 and Dma2, are involved in ubiquitination of Mmr1. In Δ*dma1* Δ*dma2* cells, Mmr1 degradation was suppressed, and mitochondria were stacked at the bud-tip and bud-neck. Serial observations of mitochondria in Δ*dma1* Δ*dma2* cells revealed that they were first stacked at the bud-tip, then moved back to the bud-neck, and again stacked together with Myo2. The backward movement of mitochondria to the bud-neck is considered a passive movement dragged by the translocation of Myo2 from the bud-tip to the bud-neck at the cytokinesis stage. Thus, mitochondria are unloaded from the myosin via the Dma1/2-mediated ubiquitination of Mmr1 and subsequent degradation by the proteasome (**Figure 1**).

Mmr1 degradation must be regulated spatiotemporally to ensure mitochondrial inheritance to daughter cells. Particularly, mitochondria should be released from myosin only after they enter the growing bud. Two protein kinases, Ste20 and Cla4, have been shown to play an important role in determining the timing and location of Mmr1 degradation. *In vitro* phosphorylation and ubiquitination assays revealed that Ste20 and Cla4 directly phosphorylate Mmr1 and that the Ste20/Cla4-mediated phosphorylation of Mmr1 is a prerequisite for its ubiquitination. The Ser414 residue of Mmr1 is most likely the key phosphorylation site for Ste20 and Cla4 in Mmr1 turnover. In Δ*mmr1* cells expressing an Mmr1-S414A mutant protein, degradation of the Mmr1-S414A protein was suppressed, and mitochondria were stacked at the bud-tip and bud-neck like in Δ*dma1* Δ*dma2* cells. Importantly, both Ste20 and Cla4 are mostly localized to the growing bud and are nearly absent from mother cells, which may be the mechanism to avoid Mmr1 degradation before mitochondria reach the growing bud.

## SIGNIFICANCE OF MITOCHONDRIAL UNLOADING FROM MYOSIN DURING BUDDING

Detailed fluorescence and electron microscopic observations revealed that stacked mitochondria in Δ*dma1* Δ*dma2* cells are often highly expanded or abnormally deformed. Therefore, unloading of mitochondria from myosin through regulated proteolysis of Mmr1 is required for the normal distribution and morphology of mitochondria. The function of mitochondria, especially stacked mitochondria, was also affected in Δ*dma1* Δ*dma2* cells. In medium containing glucose as a carbon source, yeast cells generate ATP mainly via glycolysis in the cytosol, and conversely, respiration in the mitochondria is downregulated to a low level. However, even in glucose-containing medium, Δ*dma1* Δ*dma2* cells showed elevated mitochondrial membrane potential, which roughly reflects respiration activity, suggesting the dysregulation of respiration activity in this mutant. Along with elevated mitochondrial membrane potential, Δ*dma1* Δ*dma2* cells generated more ROS than wild-type cells. Elevation of ROS levels in Δ*dma1* Δ*dma2* cells was accompanied by hypersensitivity of cells to oxidative stresses, such as ROS generating reagent paraquat and deletion of the *SOD1* gene encoding superoxide dismutase. Collectively, the regulated degradation of Mmr1 is essential for maintaining normal mitochondrial function as well as normal dynamics.

## FUTURE PERSPECTIVE

Our work demonstrated the mechanism and significance of mitochondrial unloading from myosin after their inheritance in *S. cerevisiae*. One of the most important future issues is whether this mechanism can be applied generally to other cargos in budding yeast and in higher organisms. Vacuole inheritance in yeast involves the same mechanism. During budding, vacuoles are transported to the growing bud by Myo2 on the actin cable. Dr. Weisman’s group revealed that an adaptor protein Vac17 bridging vacuoles and Myo2 is ubiquitinated by Dma1 and Dma2 and degraded by the proteasome, which is required for the release of vacuoles from Myo2 in daughter cells. Therefore, it is important to examine whether the proteolysis of adaptor proteins is a general strategy of yeast cells for unloading cargo from motor proteins. In mammalian cells, the mitochondria are often passively segregated during cell division. Interestingly, mitochondrial motor proteins dissociate from the mitochondria prior to cell division, leading to the release of mitochondria from microtubules and their passive segregation. Identifying whether proteolysis of mitochondrial motor proteins and/or adaptor proteins is involved in the “motor shedding” phenomenon before cell division would be an interesting topic for future research.

Stacked mitochondria are often highly expanded and/or deformed into an abnormally complicated morphology in Δ*dma1* Δ*dma2* cells. How mitochondrial deformation occurs in Δ*dma1* Δ*dma2* cells also needs to be determined. Mitochondrial morphology is regulated by fusion and fission. At present, it is unclear whether the expansion and deformation of mitochondria in Δ*dma1* Δ*dma2* cells are due to dysregulation of the fusion/fission balance of mitochondria. Combining deletions of genes required for mitochondrial fusion/fission with deletion of *DMA1 DMA2* would be an interesting approach to address this issue. Selective autophagy of mitochondria, known as mitophagy, is often involved in the elimination of damaged mitochondria. Monitoring mitophagy activity and examining the potential involvement of mitophagy in maintaining mitochondrial homeostasis in Δ*dma1* Δ*dma2* cells is also an intriguing future problem.

